# Understanding South Australia’s blood products usage patterns and outcomes, using data linkage.

**DOI:** 10.23889/ijpds.v7i3.1975

**Published:** 2022-08-25

**Authors:** Miro Palfy, Christopher Radbone

**Affiliations:** 1 SA NT DataLink

## Objectives

The purpose of this analytical activity was to ensure confidence in the technical capability for extracting, linking, and integrating public hospital inpatient data, public pathology blood transfusions records and blood tests, to optimise records linkage allowing patterns and trends to be then analysed with confidence.

## Approach

The SURE secure data platform was essential to ensure data governance and security requirements were met while integrating health data spanning 18 months (January 2018 - June 2019). Data sources came in multiple formats of varying quality. R was chosen for its data wrangling abilities and reproducibility.

The phases were:

1. Source data loading and cleaning
2. Linking hospital inpatient and blood transfusions records
3. Summarising linked transfusion data
4. Linking inpatient and blood tests data
5. Summarising linked tests data
6. Integrating hospital data with summarised transfusion and summarised tests data
7. Deriving additional variables based on summarised data

## Results

From 143,192 transfusion records, 55,053 (38.4%) were excluded as they did not meet the inclusion criteria (e.g., hospital or blood product out-of-scope).

From 7,897,451 blood test records, 238,013 (3.0%) were excluded, mostly of poor quality (missing/invalid hospital code).

Initially 91.4% of transfusion records were matched with hospital inpatient records. The linkage rate for state-wide blood test records was 62.3% for tests records, noting the low match rate was attributed to tests not performed on public hospital patients, as the blood test data was statewide.

Linkage process was improved by adding additional patient codes from public pathology’s internal patient identifiers. The linkage rate improved to 95.5% for transfusion records and 64.4% for test records.

## Conclusion

12 different data sources, with differing file types and formats, needed coding to achieve standardised results, enabling future reproducibility. Over one hundred business rules were implemented to produce a robust solution for future data updates. End results were analysed, and it was determined that linkage and integration quality exceeded previous similar attempts in terms of match rate and accuracy.

